# Impact of Concomitant Methotrexate Use and Prior Biologic Disease‐Modifying Antirheumatic Drug Exposure on Tofacitinib Efficacy and Safety in Patients with Polyarticular Course Juvenile Idiopathic Arthritis: Post Hoc Analysis of a Phase 3 Randomized Withdrawal Trial

**DOI:** 10.1002/acr2.70097

**Published:** 2025-10-07

**Authors:** Nicolino Ruperto, Daniel J. Lovell, Olga Synoverska, Carlos Abud‐Mendoza, Alberto Spindler, Yulia Vyzhga, Iris Reyhan, Lyudmila Grebenkina, Irit Tirosh, Lisa Imundo, Peter Chiraseveenuprapund, Daniel J Kingsbury, Betul Sozeri, Sheetal S. Vora, Sampath Prahalad, Elena Zholobova, Kevin D. Roberts, Gosford Sawyerr, Andreas Blum, Andrea Shapiro, Annette Diehl, Abbas Ebrahim, Pascal Klaus, Alberto Martini, Hermine I. Brunner, for the Paediatric Rheumatology International Trials Organisation (PRINTO) and the Pediatric Rheumatology Collaborative Study Group (PRCSG)

**Affiliations:** ^1^ Università degli Studi di Milano‐Bicocca, Milan, and Fondazione IRCCS San Gerardo dei Tintori/Paediatric Rheumatology International Trials Organisation (PRINTO) Monza Italy; ^2^ Cincinnati Children's Hospital Medical Center and University of Cincinnati Cincinnati Ohio; ^3^ Regional Children's Hospital Ivano‐Frankivsk Ukraine; ^4^ Regional Unit of Rheumatology and Osteoporosis at Central Hospital, Faculty of Medicine San Luis Potosí Mexico; ^5^ Centro Médico Privado de Reumatologia Tucumán Argentina; ^6^ Vinnytsya National Medical University N Pirogov Vinnytsya Ukraine; ^7^ Division of Rheumatology Children's Hospital Los Angeles, Keck School of Medicine, University of Southern California Los Angeles California; ^8^ Togliatti City Clinical Hospital Number 5 Togliatti Russia; ^9^ Pediatric Rheumatology Unit and Department of Pediatrics, Edmond and Lily Safra Children's Hospital, Sheba Medical Center, Tel‐HaShomer, and Sackler Faculty of Medicine, Tel Aviv University Tel Aviv Israel; ^10^ Adolescent Rheumatology Columbia University Irving Medical Center New York New York; ^11^ University of Utah Salt Lake City Utah; ^12^ Randall Children's Hospital at Legacy Emanuel Portland Oregon; ^13^ Department of Pediatric Rheumatology, Ümraniye Training and Research Hospital Istanbul Turkey; ^14^ Pediatric Rheumatology Atrium Health Levine Children's Hospital and Wake Forest University School of Medicine Charlotte North Carolina; ^15^ Departments of Pediatrics and Human Genetics Emory University School of Medicine and Children's Healthcare of Atlanta Atlanta Georgia; ^16^ Institute of Children's Health, University Children's Clinical Hospital, Sechenov University Moscow Russia; ^17^ Pfizer Inc Cambridge Massachusetts; ^18^ Pfizer Inc New York New York; ^19^ Pfizer Pharma GmbH Berlin Germany; ^20^ Pfizer Inc Pearl River New York; ^21^ Università degli Studi di Genova Genova Italy

## Abstract

**Objective:**

We evaluate tofacitinib efficacy and safety in patients with juvenile idiopathic arthritis (JIA) stratified by concomitant methotrexate and prior biologic disease‐modifying antirheumatic drug (bDMARD) treatment.

**Methods:**

In this two part, double‐blind, randomized withdrawal Phase 3 trial (NCT02592434), patients (2–<18 years) with JIA received open‐label tofacitinib (5 mg twice daily/weight‐based equivalent) in Part 1 (Day 1–Week 18). Those eligible were randomized to continue tofacitinib or switch to placebo in Part 2 (Weeks 18–44). In this post hoc analysis, patients were stratified by concomitant methotrexate (MTX+/−) and prior bDMARD treatment (bDMARD+/−). Efficacy outcomes included JIA disease flare, JIA/American College of Rheumatology (ACR) 50/70 response rates, and JIA/ACR clinical inactive disease (CID). Safety was assessed throughout the trial.

**Results:**

Of 225 patients in Part 1, 147 (65.3%) were MTX+; 85 (37.8%) were bDMARD+. Across subgroups, JIA flare rate at Week 44 was lower with tofacitinib than placebo: patients who were MTX+ (29.8% vs 47.5%), patients who were MTX− (32.3% vs 73.1%), patients who were bDMARD+ (29.0% vs 70.4%), patients who were bDMARD− (31.6% vs 48.3%). Greater JIA/ACR50/70 and JIA/ACR‐CID responses with tofacitinib than with placebo were observed across subgroups. In tofacitinib‐treated patients, adverse events (AEs) were more common in the subgroups of patients who were MTX− (85.9%) and patients who were bDMARD+ (81.2%) than the subgroups of patients who were MTX+ (74.1%) and patients who were bDMARD− (76.4%). Similar findings were observed with serious AEs/discontinuations due to AEs.

**Conclusion:**

Tofacitinib was efficacious for patients regardless of baseline concomitant MTX treatment or prior bDMARD exposure; MTX may have a protective effect for flare upon tofacitinib withdrawal. Safety was consistent with the known tofacitinib profile, and no new risks were identified in these subgroups.

## INTRODUCTION

Juvenile idiopathic arthritis (JIA) comprises several chronic inflammatory conditions of unknown etiology that begin in pediatric patients younger than 16 years of age and persist for a period of at least six weeks.^1^, ^2^, ^3^ Up to approximately 50% of patients experience a more severe JIA disease course, termed polyarticular course (pc) JIA,^4^ which is a functional concept that includes the following JIA categories: extended oligoarthritis, rheumatoid factor (RF)‐positive or RF‐negative polyarthritis, and systemic (s) JIA without systemic features at enrollment.^2^ Other categories of JIA include enthesitis‐related arthritis (ERA) and juvenile psoriatic arthritis (jPsA).^1^

The American College of Rheumatology (ACR)/Arthritis Foundation guidelines recommend that patients with pcJIA are initially treated with conventional synthetic disease‐modifying antirheumatic drugs (csDMARDs), typically methotrexate (MTX).^5^ In patients who continue to have moderate to high disease activity, treatment with csDMARDs is escalated to biologic (b) DMARDs, such as tumor necrosis factor inhibitors (TNFis),^5^ or other therapies with alternative mechanisms of action. Indeed, approximately a third of patients with JIA fail to respond to MTX,^6^ and MTX therapy for patients with JIA is also associated with certain side effects such as nausea and liver toxicity,^7^ leaving many patients with difficulty taking this therapy.^8^ Although bDMARD treatment has significantly improved outcomes for patients with JIA,^9^, ^10^, ^11^ refractory disease to bDMARD therapy can occur, and in some patients who were unsuccessful with prior bDMARD treatments, they may have a reduced response to subsequent biologic treatments, as noted in randomized controlled trials.^10^, ^11^ In patients who have achieved adequate disease control, reducing medication exposure (eg, by treating with monotherapy) may be considered, to potentially decrease the risk of treatment‐related AEs.^12^, ^13^

Tofacitinib is an oral JAK inhibitor indicated for the treatment of patients with pcJIA and patients with jPsA and is under investigation for patients with sJIA. In this post hoc analysis of a double‐blind, placebo‐controlled, Phase 3 randomized withdrawal trial, whose primary results were reported previously,^14^ we assessed the efficacy and safety of tofacitinib in patients with JIA, stratified by concomitant MTX use at baseline and prior exposure to bDMARDs.

## PATIENTS AND METHODS

### Study design

This was a Phase 3, two‐part, randomized withdrawal trial (NCT02592434) conducted at centers of the Pediatric Rheumatology International Trials Organisation (PRINTO)^15^ and Pediatric Rheumatology Collaborative Study Group (PRCSG) (Appendix A).^16^ Full study design details have been reported previously.^14^ Briefly, the trial consisted of an open‐label phase (Part 1) and a randomized, double‐blind, placebo‐controlled phase (Part 2). Eligible patients were aged 2–<18 years and had one of the following forms of JIA per the International League of Associations for Rheumatology classification criteria:^1^ extended oligoarthritis, RF‐positive or RF‐negative polyarthritis and sJIA without systemic features (hereafter collectively termed pcJIA) for 6 months before enrollment. Patients with jPsA and ERA were included for exploratory assessment of tofacitinib in these JIA categories. Patients with pcJIA were required to have active disease, and an inadequate response to DMARDs (MTX or bDMARD). Patients with jPsA or ERA were required to have active disease (defined as ≥3 active joints at enrollment), and an inadequate response to nonsteroidal anti‐inflammatory drugs (NSAIDs).

In Part 1 (open‐label run‐in phase; Day 1–Week 18), patients received tofacitinib 5 mg twice daily (BID) as tablets or a bodyweight‐based lower equivalent dose of oral solution (1 mg/mL) until Week 18 or JIA flare. In Part 2 (double‐blind phase, Weeks 18–44), patients achieving a JIA/ACR30 response (ie, an improvement of ≥30% in three of the six JIA core set variables) at Week 18 of Part 1 were randomized 1:1 to continue receiving tofacitinib or switch to placebo for 26 weeks or until JIA flare. Patients who experienced a JIA flare at any time during the trial (Part 1 or Part 2) were withdrawn from the trial. Stable doses of NSAIDs, MTX (at doses not to exceed 25 mg per week or 20 mg/m^2^ per week, whichever was lower) and oral glucocorticoids (at a maximum dose of 0.2 mg/kg/day of prednisone equivalent or ≤10 mg/day, whichever was lower) could be continued throughout the trial.^14^

The trial was conducted in accordance with the Guidelines for Good Clinical Practice, the Declaration of Helsinki, and local regulatory requirements and laws, and was approved by Institutional Review Boards or Ethics Committees at all sites. Written informed consent or assent was provided by parents, legal guardians, or patients. The Consolidated Standards of Reporting Trials (CONSORT)^17^, ^18^, ^19^ were followed.

### Post hoc analysis assessing the impact of concomitant MTX and prior bDMARD treatment on tofacitinib efficacy and safety

This post hoc analysis included patients with pcJIA, jPsA, or ERA. The patient population was stratified by the following subgroups: (1) concomitant MTX use on trial Day 1 (yes/no [termed MTX+ or MTX−]) and (2) patients with prior bDMARD exposure (yes/no [termed bDMARD+ or bDMARD−]). Analyses of specific endpoints were also performed on the following additional patient subgroups: MTX+/bDMARD−, MTX+/bDMARD+, MTX−/bDMARD+, and MTX−/bDMARD−. Stratification by prior MTX treatment was not conducted; 90.7% of the patients had prior MTX treatment.

### Efficacy assessments

Efficacy for tofacitinib versus placebo was assessed by subgroup for the following outcomes: JIA flare rate at the end of Part 2 (Week 44), defined per PRCSG and PRINTO JIA flare criteria^20^ as a worsening of ≥30% in at least three of six JIA/ACR core set variables, with one or none of the variables improving by ≥30%; time to disease flare in Part 2; JIA/ACR50 and JIA/ACR70 response rates (≥50/70% improvement in ≥3 of 6 JIA/ACR core set variables)^21^ in Part 1 and Part 2; JIA/ACR clinical inactive disease (CID) rates^22^, ^23^ in Part 1 and Part 2; mean juvenile arthritis disease activity score in 27 joints using C‐reactive protein (JADAS)^24^, ^25^ in Part 1 and Part 2; and mean change from baseline in Childhood Health Assessment Questionnaire ‐ Disability Index (CHAQ‐DI) responses^26^, ^27^ at the end of Part 2 (Week 44). For the analyses by the additional patient subgroups, JIA flare rate at the end of Part 2 (Week 44); JIA/ACR50, JIA/ACR70, and JIA/ACR‐CID rates in Part 1 and Part 2; and mean change from Part 2 baseline in CHAQ‐DI at Week 44 were assessed.

### Safety assessments

Safety of tofacitinib was assessed in all patient subgroups throughout the duration of the trial; outcomes included: adverse events (AEs), serious AEs, AEs of special interest (AESIs), discontinuations due to AEs, and laboratory abnormalities. AESIs included hepatic events, herpes zoster (serious and nonserious) and serious infections, opportunistic infections (including tuberculosis), malignancies, macrophage activation syndrome, major adverse cardiovascular events, gastrointestinal perforations, interstitial lung disease, and thrombotic events. Events were coded using the Medical Dictionary for Regulatory Activities (MedDRA) version 22.0.

### Statistical analysis

In the analyses of binary endpoints, including JIA flare rate, JIA/ACR50 and JIA/ACR70 response rates, and JIA/ACR‐CID rates, standard errors were based on the normal distribution approximation to the binomial. JIA flare was measured based on disease course relative to JIA status at the time of randomization upon entry into Part 2, whereas JIA/ACR50 and JIA/ACR70 response rates were calculated relative to Part 1 baseline. If a patient discontinued trial treatment in Part 2 because of a flare or any other reason except for clinical remission (≥24 consecutive weeks of inactive disease), that patient was considered to have active disease and classified as a JIA/ACR nonresponder, as per the intention‐to‐treat principle.^17^, ^18^, ^19^ All the above analyses were completed with the data as observed. No imputations were conducted for missing data.

Continuous variables (JADAS and CHAQ‐DI) were summarized using descriptive statistics for observed values and changes from baseline or Part 2 baseline. A mixed‐effect linear model with repeated measures using observed data was performed for JADAS comparing tofacitinib with placebo during Part 2. Kaplan‐Meier estimates of the survivor functions from time to JIA flare were obtained. No formal statistical hypothesis testing was conducted.

Upon request, and subject to review, Pfizer will provide the data that support the findings of this study. Subject to certain criteria, conditions and exceptions, Pfizer may also provide access to the related individual de‐identified participant data. See https://www.pfizer.com/science/clinical‐trials/trial‐data‐and‐results for more information.

## RESULTS

### Patients

Overall, 225 patients with pcJIA, jPsA, or ERA entered Part 1 of the study; 173 were randomized to receive tofacitinib (n = 88) or placebo (n = 85) in Part 2, and 99 completed Part 2 (tofacitinib, n = 61; placebo, n = 38). Insufficient clinical response was the most common reason for discontinuation in both arms during Part 2 (tofacitinib, 22 of 88 [25.0%]; placebo, 44 of 85 [51.8%]). Of the 225 patients who received treatment with tofacitinib in Part 1, 147 (65.3%) received concomitant treatment with MTX (MTX+), and 85 (37.8%) had prior bDMARD exposure (bDMARD+) (Table 1). In the additional patient subgroups, 38 (16.9%) were MTX+ and bDMARD+, 109 (48.4%) were MTX+ and bDMARD−, 47 (20.9%) were MTX− and bDMARD+, and 31 (13.8%) were MTX− and bDMARD− (Supplementary Table 1). Overall, 90.7% (204 of 225) of patients had previously received MTX. Across subgroups, most patients receiving tofacitinib in Part 1 were female (>72%), median age ranged from 12.0–13.0 years, and >50% of patients were aged 12–<18 years (Table 1).

**Table 1:** Demographics and disease characteristics of patients with pcJIA, jPsA, and ERA in Part 1*

	Tofacitinib 5 mg BID^b^ (n = 225)				
Part 1^a^	MTX+ (n = 147)	MTX− (n = 78)	Prior bDMARD+ (n = 85)	Prior bDMARD− (n = 140)	All patients (N = 225)
Female, n, %	110 (74.8)	59 (75.6)	62 (72.9)	107 (76.4)	169 (75.1)
Age, y					
Median (Q1–Q3)	12.0 (8.0–15.0)	13.0 (11.0–15.0)	13.0 (11.0–15.0)	13.0 (8.0–16.0)	13.0 (9.0–15.0)
2–<6 years, n (%)	18 (12.2)	4 (5.1)	4 (4.7)	18 (12.9)	22 (9.8)
6–<12 years, n (%)	46 (31.3)	18 (23.1)	25 (29.4)	39 (27.9)	64 (28.4)
12–<18 years, n (%)	83 (56.5)	56 (71.8)	56 (65.9)	83 (59.3)	139 (61.8)
Disease duration, y					
Median (Q1–Q3)	2.0 (1.0–4.3)	4.4 (1.7–8.0)	5.1 (2.4–8.2)	1.7 (0.7–3.9)	2.5 (1.0–5.6)
JIA core set measures					
Physician's global evaluation of overall disease activity,^c^ median (Q1–Q3)	6.0 (4.5–7.5)	6.0 (5.0–8.0)	6.5 (5.0–8.5)	6.0 (4.5–7.5)	6.0 (4.5–7.5)
Number of joints with active arthritis,^d^ median (Q1–Q3)	9.0 (6.0–14.0)	11.0 (6.0–18.0)	13.0 (8.0–19.0)	8.0 (6.0–12.0)	10.0 (6.0–15.0)
Number of joints with limitation of motion, median (Q1–Q3)	5.0 (2.0–9.0)	6.5 (4.0–12.0)	8.0 (4.0–15.0)	5.0 (2.0–8.0)	6.0 (3.0–10.0)
CHAQ‐DI scores,^e^ median (Q1–Q3)	0.9 (0.4–1.6)	0.8 (0.3–1.4)	1.1 (0.5–1.6)	0.8 (0.3–1.4)	0.9 (0.3–1.5)
Patient overall well‐being, median (Q1–Q3)	5.0 (3.0–7.0)	5.0 (3.0–7.0)	5.0 (3.5–7.5)	5.0 (3.0–6.5)	5.0 (3.0–7.0)
CRP,^f^ mg/dL, median (Q1–Q3)	0.2 (0.1–1.1)	0.3 (0.1–0.9)	0.4 (0.1–1.8)	0.2 (0.1–0.8)	0.3 (0.1–1.0)
ESR,^g^ mm/h, median (Q1–Q3)	18.0 (10.0–29.0)	16.0 (9.0–35.0)	20.0 (10.0–40.0)	16.0 (10.0–28.0)	17.0 (10.0–32.0)
JADAS,^h^ median (Q1–Q3)	20.1 (16.7–26.7)	19.6 (15.2–26.5)	23.6 (16.6–32.0)	19.1 (15.7–27.8)	20.1 (16.2–26.6)
Duration of morning stiffness, minutes, median (Q1–Q3)	30.0 (16.0–60.0)	30.0 (15.0–90.0)	40.0 (20.0–90.0)	30.0 (10.0–60.0)	30.0 (15.0–60.0)

* Some of the data for all patients have previously been published.^14^ bDMARD, biologic disease‐modifying antirheumatic drug; BID, twice daily; CHAQ‐DI, Childhood Health Assessment Questionnaire‐Disability Index; CRP, C‐reactive protein; ERA, enthesitis‐related arthritis; ESR, erythrocyte sedimentation rate; JADAS, juvenile arthritis disease activity score in 27 joints using C‐reactive protein; JIA, juvenile idiopathic arthritis; jPsA, juvenile psoriatic arthritis; MTX, methotrexate; pcJIA, polyarticular course juvenile idiopathic arthritis; Q, quartile.

^a^ Open‐label run‐in phase (Day 1–Week 18).

^b^ Tofacitinib 5 mg BID or equivalent weight‐based lower dose in patients <40 kg.

^c^ Scores could range from 0–10, with higher scores indicating more disease activity.

^d^ Active arthritis defined as any joint with swelling, or in the absence of swelling, limitation of motion accompanied by either pain on motion or tenderness not due to deformity.

^e^ Scores could range from 0–3, with higher scores indicating more disability.

^f^ Normal reference range was 0–0.287 mg/dL.

^g^ Normal reference range was 0–20 mm/h.

^h^ Scores could range from 0–57, with higher scores indicating more disease activity.

Generally, baseline disease characteristics of patients receiving tofacitinib in Part 1 were similar across subgroups (Table 1), although some differences were observed. Patients receiving tofacitinib in the MTX+ subgroup had shorter median disease duration (2.0 vs 4.4 years), and a lower median number of joints with active arthritis (9.0 vs 11.0), and joints with limitation of motion (5.0 vs 6.5) at baseline than patients in the MTX− group. Patients receiving tofacitinib in the bDMARD+ subgroup had longer disease duration (5.1 years vs 1.7 years), a higher number of joints with active arthritis (13.0 vs 8.0) and joints with limitation of motion (8.0 vs 5.0), higher CHAQ‐DI scores (1.1 vs 0.8), erythrocyte sedimentation rate (20.0 vs 16.0), JADAS (23.6 vs 19.1), and a longer duration of morning stiffness (40.0 minutes vs 30.0 minutes) at baseline than patients in the bDMARD− subgroup (Table 1).

### JIA flare rate and time to disease flare

**Figure 1:**
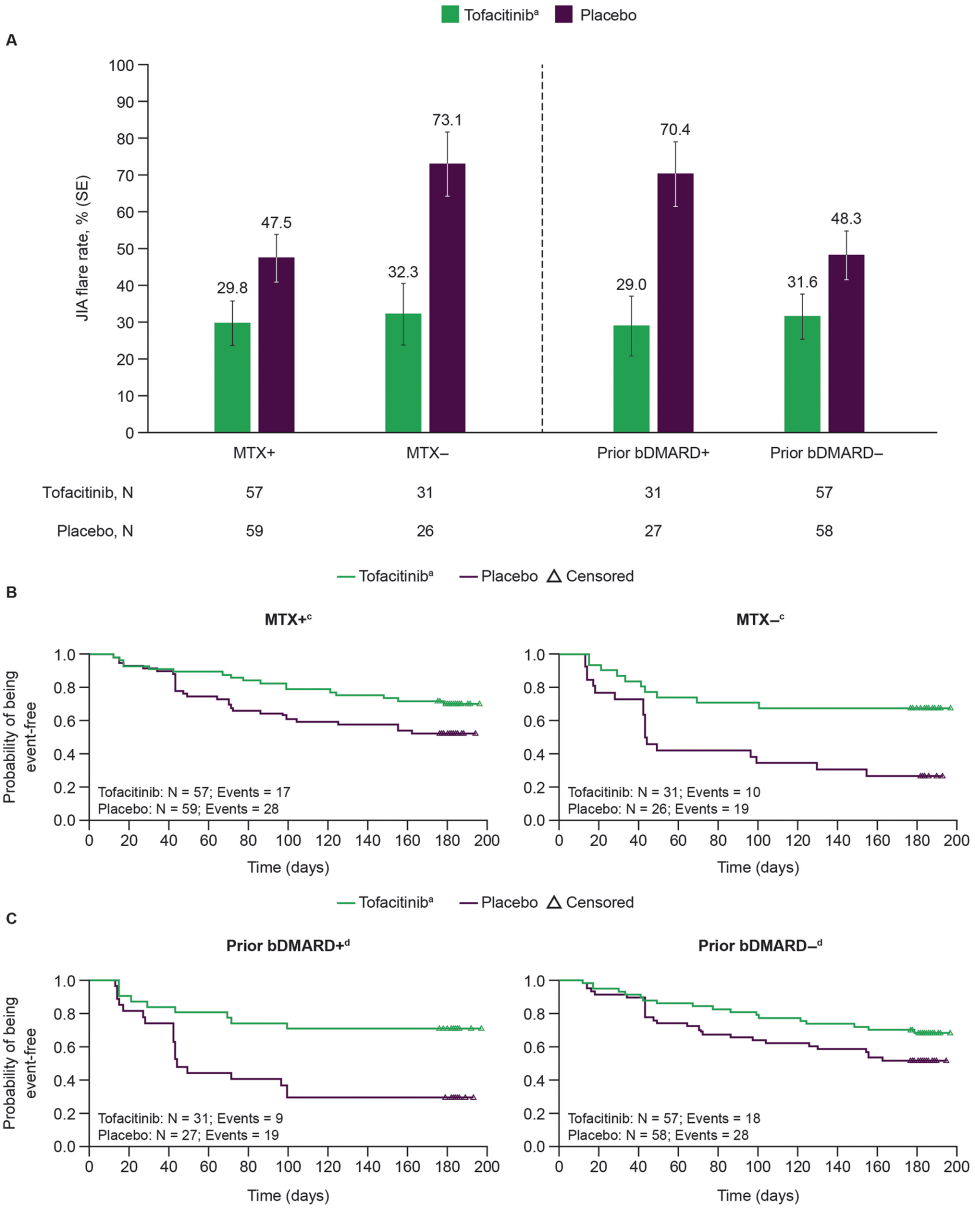
(A) JIA flare rate (SE) by Week 44 in patients receiving tofacitinib^a^ or placebo stratified by concomitant MTX treatment or prior bDMARD exposure and Kaplan‐Meier plots of time to disease flare in Part 2^b^ stratified by (B) concomitant MTX treatment and (C) prior bDMARD exposure. ^a^Tofacitinib 5 mg BID or equivalent weight‐based lower dose in patients weighing <40 kg. ^b^Part 2, the double‐blind phase, is the trial period on and after randomization day (Weeks 18–44). Time to disease flare was measured in the number of days since randomization (day of first disease flare to day of randomization + 1) into the double‐blind phase up to Day 196. ^c^Median time to disease flare could not be calculated for patients treated with tofacitinib in either MTX subgroup or for patients receiving placebo with concomitant MTX because >50% remained flare‐free. Median time to disease flare (95% CI) was 43.5 days (42.0–129.0) for patients receiving placebo without concomitant MTX. ^d^Median time to disease flare could not be calculated for patients treated with tofacitinib in either bDMARD subgroup or for patients receiving placebo without prior bDMARD exposure because >50% remained flare‐free. Median time to disease flare (95% CI) was 44.0 days (42.0–99.0) for patients receiving placebo with prior bDMARD exposure. bDMARD, biologic disease‐modifying antirheumatic drug; BID, twice daily; CI, confidence interval; JIA, juvenile idiopathic arthritis; MTX, methotrexate.

In Part 2 of the trial, within each subgroup, JIA flare rate by Week 44 was lower with tofacitinib treatment compared with placebo following tofacitinib withdrawal: MTX+ (17 of 57 [29.8%] vs 28 of 59 [47.5%]), MTX− (10 of 31 [32.3%] vs 19 of 26 [73.1%]), bDMARD+ (9 of 31 [29.0%] vs 19 of 27 [70.4%]), bDMARD− (18 of 57 [31.6%] vs 28 of 58 [48.3%]). JIA flare rates with tofacitinib were similar between the groups who were MTX+ or MTX− and the groups who were bDMARD+ or bDMARD−, whereas those with placebo were greater for patients in the MTX− subgroup than patients in the MTX+ subgroup (19 of 26 [73.1%] vs 28 of 59 [47.5%], respectively), and for patients in the bDMARD+ than in the bDMARD− subgroup (19 of 27 [70.4%] vs 28 of 58 [48.3%], respectively) (Figure 1A). In addition, JIA flare rates by Week 44 were lower with tofacitinib than with placebo when patients were stratified by additional patient subgroups of concomitant MTX and prior bDMARD exposure (Supplementary Figure 1).

Kaplan‐Meier plots of the time to disease flare in Part 2 are shown in Figure 1B and C. Regardless of concomitant MTX use or prior bDMARD exposure, patients who received tofacitinib had longer time to disease flare than those who received placebo. The difference in time to disease flare was greater between tofacitinib and placebo in the MTX− subgroup than in the MTX+ subgroup, and in the bDMARD+ subgroup, than in the bDMARD− subgroup. (Figure 1B and C).

### Treatment response and CID

**Figure 2:**
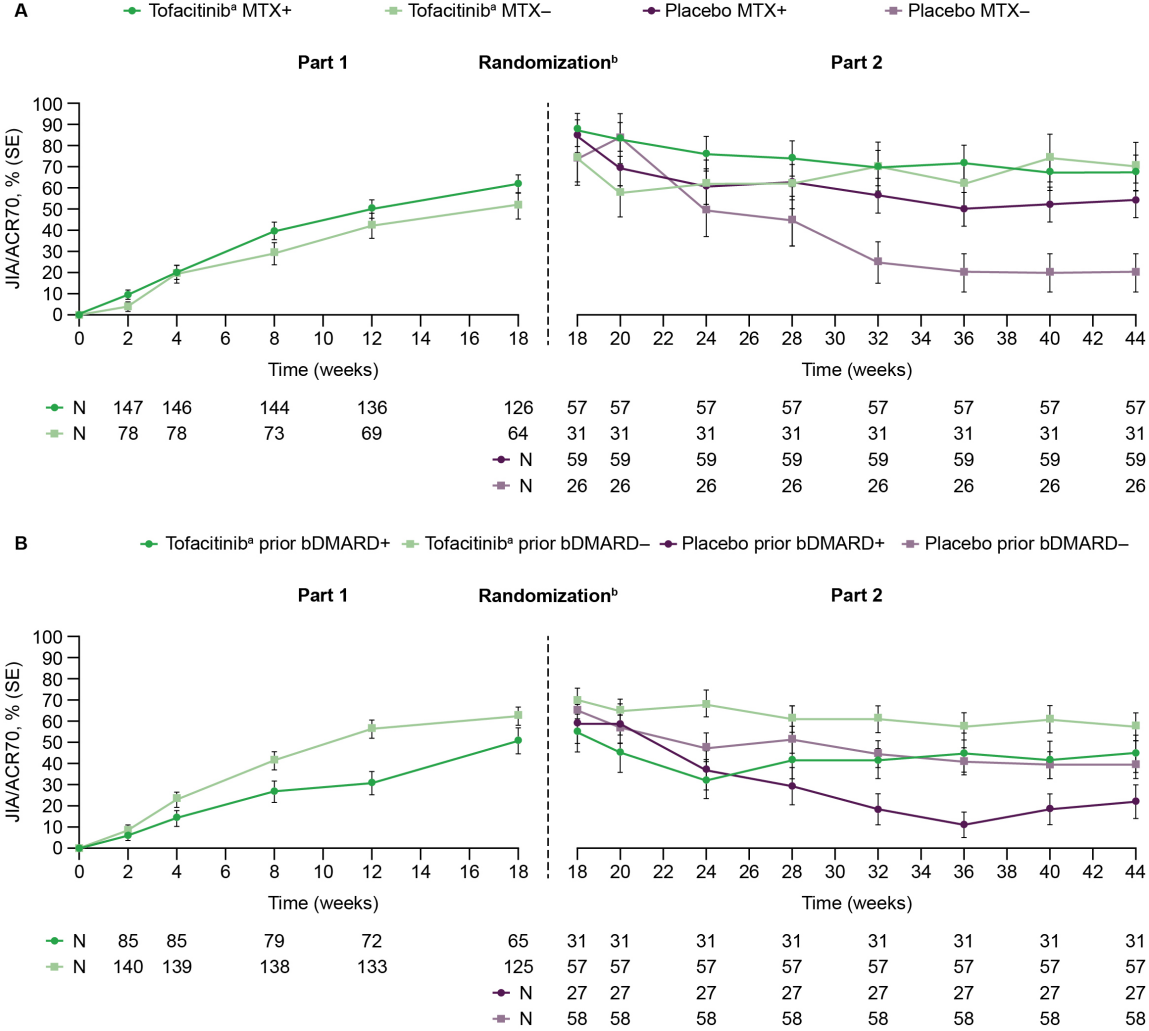
Response rates (SE) over time in patients receiving tofacitinib^a^ or placebo stratified by (A) concomitant MTX treatment and (B) prior bDMARD exposure for JIA/ACR70. ^a^Tofacitinib 5 mg BID or equivalent weight‐based lower dose in patients weighing <40 kg. ^b^In Part 1 (Weeks 0–18), all patients received open‐label tofacitinib. Patients achieving ≥JIA/ACR30 response at Week 18 were blindly randomized 1:1 to receive tofacitinib or placebo in Part 2 (Weeks 18–44). Patients who discontinued during Part 2 because of flare or any other reason except for clinical remission (≥24 consecutive weeks of inactive disease), were considered to have active disease and classified as non‐responders, as per the intention‐to‐treat principle.^17^, ^18^, ^19^ bDMARD, biologic disease‐modifying antirheumatic drug; BID, twice daily; JIA/ACR30/70, juvenile idiopathic arthritis/American College of Rheumatology 30/70 response; MTX, methotrexate.

In Part 1, JIA/ACR50 and JIA/ACR70 response rates and JIA/ACR‐CID rates improved over time in all subgroups of patients receiving tofacitinib, and similar trends in response rates over time were observed between patients in the MTX+ and MTX− subgroups, patients in the bDMARD+ and bDMARD− subgroups, and the additional patient subgroups (Figure 2 and Supplementary Figure 2 and 3). In Part 2, across patient subgroups, JIA/ACR50 and JIA/ACR70 response rates and JIA/ACR‐CID rates were generally stable in patients who continued receiving tofacitinib; scores tended to worsen with placebo over time, particularly in patients in the MTX− subgroup, the bDMARD+ subgroup, and the MTX−/bDMARD+ additional subgroup (Figure 2 and Supplementary Figure 2 and 3).

At Week 44, across patient subgroups, JIA/ACR50 and JIA/ACR70 response rates and JIA/ACR‐CID rates were generally higher with tofacitinib than placebo, with the greatest differences in these respective rates generally observed between tofacitinib and placebo in patients in the MTX− and bDMARD+ subgroups (Figure 2 and Supplementary Figure 2).

### Disease activity

**Figure 3:**
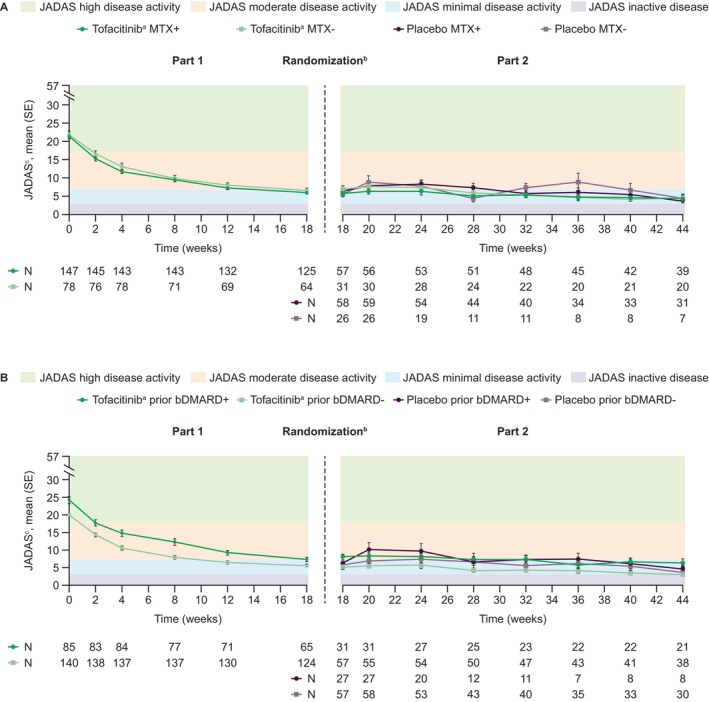
Mean (SE) JADAS over time in patients receiving tofacitinib^a^ or placebo stratified by (A) concomitant MTX treatment and (B) prior bDMARD exposure. Observed case analysis. ^a^Tofacitinib 5 mg BID or equivalent weight‐based lower dose in patients weighing <40 kg. ^b^In Part 1 (Day 1–Week 18), all patients received open‐label tofacitinib. Patients achieving ≥JIA/ACR30 response at Week 18 were blindly randomized 1:1 to receive tofacitinib treatment or placebo in Part 2 (Weeks 18–44). ^c^Scores could range from 0−57, with higher scores indicating greater disease activity. Inactive disease, minimal disease activity, moderate disease activity, and high disease activity were defined as a JADAS score of ≤2.7, 2.8–6, 6.1–17, and >17, respectively,^36^ indicated by the shaded areas. bDMARD, biologic disease‐modifying antirheumatic drug; BID, twice daily; JADAS, juvenile arthritis disease activity score in 27 joints using C‐reactive protein; JIA/ACR30, juvenile idiopathic arthritis/American College of Rheumatology 30 response; MTX, methotrexate.

JADAS improved with tofacitinib treatment during Part 1, with similar improvements noted for patients in the MTX+/− and bDMARD+/− subgroups. In Part 2, mean JADAS scores were generally more stable in patients receiving tofacitinib than with placebo and were consistent among subgroups (Figure 3).

**Figure 4:**
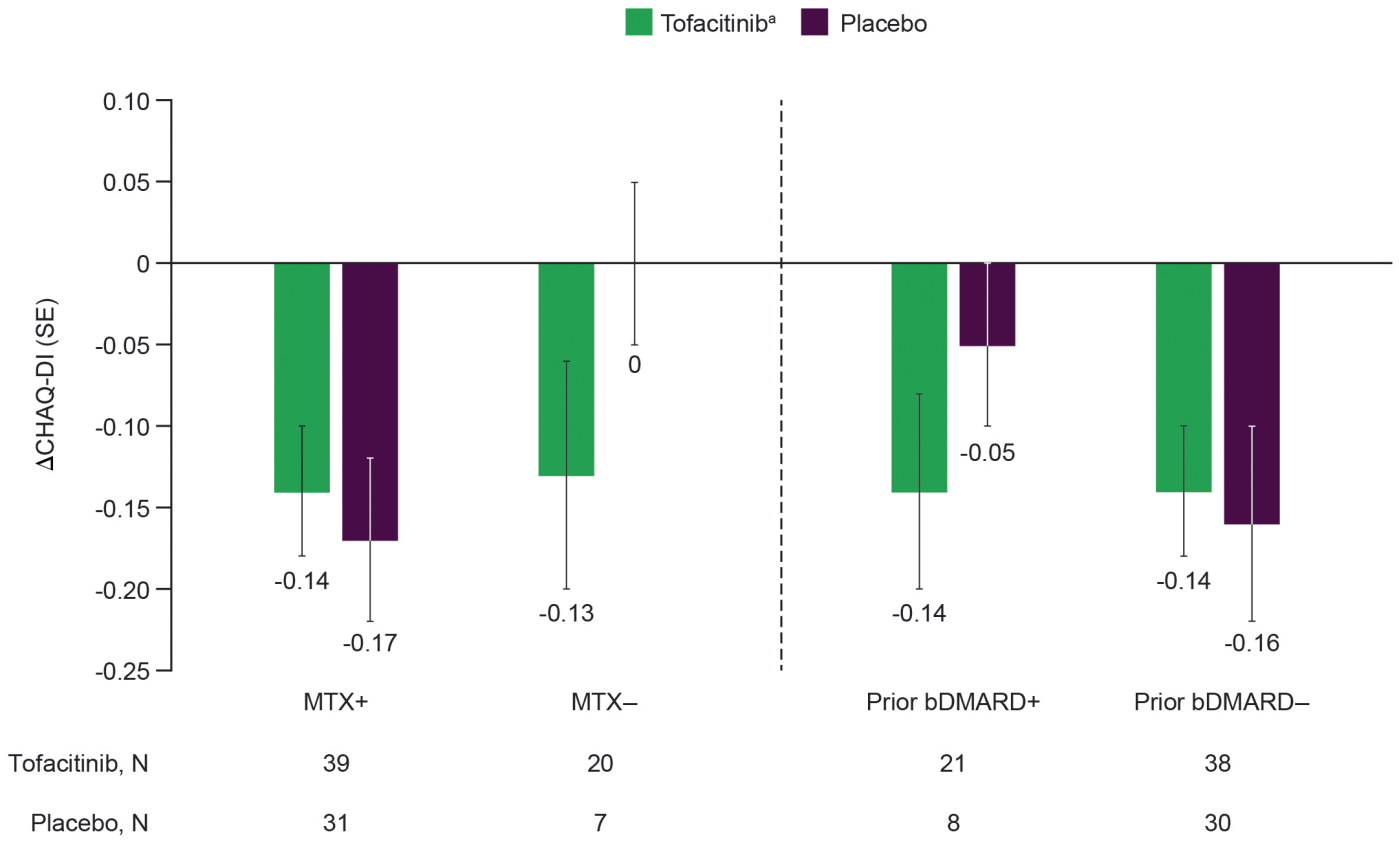
Mean (SE) change from baseline^a^ in CHAQ‐DI at Week 44 in patients receiving tofacitinib^b^ treatment or placebo stratified by concomitant MTX treatment and prior bDMARD exposure. Observed case analysis. ^a^Week 18 was used as baseline. ^b^Tofacitinib 5 mg BID or equivalent weight‐based lower dose in patients weighing <40kg. ΔCHAQ‐DI, change from baseline in Childhood Health Assessment Questionnaire – Disability Index; bDMARD, biologic disease‐modifying antirheumatic drug; BID, twice daily; MTX, methotrexate.

By Week 44, physical functioning, as assessed by CHAQ‐DI, improved in patients treated with tofacitinib, and at a similar magnitude between subgroups (Figure 4). In patients treated with placebo in Part 2, improvements from randomization were observed in those in the MTX+ and bDMARD+/− subgroups at a generally similar magnitude to the respective subgroups with tofacitinib (overlapping error bars). No improvement with placebo was observed in the MTX− subgroup (Figure 4). Across the additional subgroups, improvements were observed for patients treated with tofacitinib, while improvements with placebo in Part 2 were only made in MTX+ subgroups (Supplementary Figure 4).

### Safety

Across the entire tofacitinib exposure period, AEs were more common in patients receiving tofacitinib in the MTX− subgroup (67 of 78 [85.9%]) than patients in the MTX+ subgroup (109 of 147 [74.1%]), and patients in the bDMARD+ subgroup (69 of 85 [81.2%]) than patients in the bDMARD− subgroup (107 of 140 [76.4%]), whereas patients who had serious AEs were similar across subgroups (2.9%–4.7%). Of note, AEs in relation to lack of efficacy (condition aggravated, disease progression, and JIA worsening) were more frequent in patients in the MTX− (14 of 78 [17.9%]) and patients in the bDMARD+ (15 of 85 [17.6%]) subgroups, than in patients in the MTX+ (13 of 147 [8.8%]) and patients in the bDMARD− (12 of 140 [8.6%]) subgroups, respectively. A higher proportion of discontinuations due to AEs were observed in patients receiving tofacitinib in the MTX− subgroup compared with patients in the MTX+ subgroup (19 of 78 [24.4%] vs 23 of 147 [15.6%], respectively), and patients in the bDMARD+ compared with patients in the bDMARD− subgroup (21 of 85 [24.7%] vs 21 of 140 [15.0%], respectively). Temporary dose reductions or holds due to AEs were similar between subgroups (9.4–12.1%; Table 2).

**Table 2:** Safety events in the entire tofacitinib exposure period (Part 1 and Part 2) stratified by concomitant MTX use and prior bDMARD exposure*

Patients with events, n (%)	Tofacitinib^a^ MTX+ (n = 147)	Tofacitinib^a^ MTX− (n = 78)	Tofacitinib^a^ prior bDMARD+ (n = 85)	Tofacitinib^a^ prior bDMARD− (n = 140)
AEs	109 (74.1)	67 (85.9)	69 (81.2)	107 (76.4)
Serious AEs	5 (3.4)	3 (3.8)	4 (4.7)	4 (2.9)
Deaths	0	0	0	0
Permanent discontinuation due to AEs	23 (15.6)	19 (24.4)	21 (24.7)	21 (15.0)
Temporary dose reductions or temporary hold due to AEs	17 (11.6)	8 (10.3)	8 (9.4)	17 (12.1)
Most common AEs by preferred term (>5% of any subgroup)				
Abdominal pain	4 (2.7)	4 (5.1)	5 (5.9)	3 (2.1)
Back pain	3 (2.0)	5 (6.4)	3 (3.5)	5 (3.6)
Condition aggravated	1 (0.7)	4 (5.1)	3 (3.5)	2 (1.4)
Decreased appetite	1 (0.7)	5 (6.4)	1 (1.2)	5 (3.6)
Diarrhea	2 (1.4)	4 (5.1)	3 (3.5)	3 (2.1)
Disease progression	7 (4.8)	6 (7.7)	8 (9.4)	5 (3.6)
Epistaxis	3 (2.0)	3 (3.8)	5 (5.9)	1 (0.7)
Headache	8 (5.4)	10 (12.8)	6 (7.1)	12 (8.6)
Increased ALT levels	8 (5.4)	1 (1.3)	4 (4.7)	5 (3.6)
Increased AST levels	9 (6.1)	2 (2.6)	4 (4.7)	7 (5.0)
Increased blood creatine phosphokinase	3 (2.0)	4 (5.1)	1 (1.2)	6 (4.3)
Influenza	7 (4.8)	3 (3.8)	3 (3.5)	7 (5.0)
JIA worsening	5 (3.4)	4 (5.1)	4 (4.7)	5 (3.6)
Nasopharyngitis	8 (5.4)	7 (9.0)	7 (8.2)	8 (5.7)
Nausea	13 (8.8)	1 (1.3)	3 (3.5)	11 (7.9)
Oropharyngeal pain	2 (1.4)	4 (5.1)	4 (4.7)	2 (1.4)
Pyrexia	8 (5.4)	6 (7.7)	4 (4.7)	10 (7.1)
Sinusitis	4 (2.7)	4 (5.1)	2 (2.4)	6 (4.3)
Vomiting	7 (4.8)	6 (7.7)	5 (5.9)	8 (5.7)
Upper respiratory tract infection	18 (12.2)	16 (20.5)	10 (11.8)	24 (17.1)
AESI				
Hepatic events^b^	3 (2.0)	0	0	3 (2.1)
Herpes zoster (nonserious and serious)^c^	2 (1.4)	0	1 (1.2)	1 (0.7)
Serious infections^d^	2 (1.4)	1 (1.3)	2 (2.4)	1 (0.7)
Laboratory abnormalities				
AST >3.0 × ULN	4 (2.7)	0	1 (1.2)	3 (2.1)
ALT >3.0 × ULN	6 (4.1)	0	1 (1.2)	5 (3.6)
Hemoglobin <0.8 × LLN	2 (1.4)^e^	0	1 (1.2)^f^	1 (0.7)
Lymphocytes				
<0.8 × LLN	11 (7.5)^e^	0	1 (1.2)^f^	10 (7.1)
>1.2 × ULN	3 (2.1)^e^	0	2 (2.4)^f^	1 (0.7)

* AE, adverse event; AESI, adverse events of special interest; ALT, alanine aminotransferase; AST, aspartate aminotransferase; bDMARD, biologic disease‐modifying antirheumatic drug; BID, twice daily; JIA, juvenile idiopathic arthritis; LLN, lower limit of normal; MTX, methotrexate; ULN, upper limit of normal.

^a^ Tofacitinib 5 mg BID or equivalent weight‐based lower dose in patients weighing <40 kg.

^b^ All events were adjudicated; two events had a >50%–75% certainty, and one event had a >25%–50% certainty of having hepatocellular drug induced liver injury. Two events were mild, and one event was moderate.

^c^ The two patients who had herpes zoster infections had both mild, monodermatomal and nonserious infections, and neither met opportunistic infection criteria. One of the patients had never had chicken pox and had received the chicken pox vaccine. The other patient had previously had chicken pox and had not received the chicken pox vaccine. Neither patient had previously had herpes zoster, and neither had received the herpes zoster vaccine.

^d^ Serious infections were reported in three patients: one with pneumonia, one with epidural empyema and sinusitis in a patient with a history of craniosynostosis repair, and one with appendicitis.

^e^ n = 146.

^f^ n = 84.

The most common AEs, by MedDRA preferred term, were upper respiratory tract infections in all subgroups (11.8%–20.5%). A numerically higher proportion of patients in the MTX+ subgroup than patients in the MTX− subgroup experienced increased alanine aminotransferase (ALT; 8 of 147 [5.4%] vs 1 of 78 [1.3%]) levels and aspartate aminotransferase (AST; 9 of 147 [6.1%] vs 2 of 78 [2.6%]) levels. In addition, a higher proportion of patients in the MTX− (4 of 78 [5.1%]) and patients in the bDMARD− (6 of 140 [4.3%]) subgroups experienced increased blood creatine phosphokinase levels than patients in the MTX+ (3 of 147 [2.0%]) and bDMARD+ (1 of 85 [1.2%]) subgroups, respectively (Table 2).

AESIs occurred at a low frequency across subgroups. For example, hepatic events occurred in three patients each in the MTX+ (2.0%) and bDMARD− (2.1%) subgroups; herpes zoster events were reported in two patients in the MTX+ (1.4%) subgroup and one patient each in the bDMARD+ (1.2%) and bDMARD− (0.7%) subgroups; serious infections were reported in two patients each in the MTX+ (1.4%) and bDMARD+ (2.4%) subgroups and one patient each in the MTX− (1.3%) and bDMARD− (0.7%) subgroups. There were no deaths (Table 2), and there were no opportunistic infections (including tuberculosis), malignancies, macrophage activation syndrome, major adverse cardiovascular events, gastrointestinal perforations, interstitial lung disease, or thrombotic events.

No patients in the MTX− subgroup experienced laboratory abnormalities, whereas four of 147 (2.7%) patients and six of 147 (4.1%) patients in the MTX+ subgroup experienced elevated levels of AST and ALT (>3.0 × upper limit of normal) respectively; 11 of 146 (7.5%) and 10 of 140 (7.1%) patients in the MTX+ and bDMARD− subgroups experienced low levels of lymphocytes (<0.8 × lower limit of normal), respectively. Generally, a higher proportion of patients receiving tofacitinib in the bDMARD− subgroup than patients in the bDMARD+ subgroup exhibited laboratory abnormalities (Table 2). A similar rate of AEs was observed when patients were stratified by the additional patient subgroups (Supplementary Table 1).

## DISCUSSION

In this post hoc analysis, we used data from a two‐part, Phase 3 randomized withdrawal trial to assess the efficacy and safety of tofacitinib in patients with JIA, stratified by concomitant MTX treatment at baseline and prior exposure to bDMARDs. The findings of this analysis suggest that tofacitinib was efficacious irrespective of concomitant MTX treatment or prior bDMARD exposure in patients with JIA. In the open‐label phase (Part 1), tofacitinib improved JIA/ACR response rates and disease activity, whereas in the withdrawal period (Part 2), flare rates were lower with tofacitinib compared with placebo, regardless of subgroup analyzed. In addition, flare rates for placebo following tofacitinib withdrawal were greater for patients in the MTX− and bDMARD+ subgroups compared with patients in the MTX+ and bDMARD− subgroups, respectively.

In Part 1 (Day 1–Week 18), across outcomes, tofacitinib efficacy was demonstrated, and was consistent between patients in the MTX+/− and in the bDMARD+/− subgroups. In Part 2 (Weeks 18–44), tofacitinib efficacy was generally consistent across subgroups, although it tended to be greater in the bDMARD− than in the bDMARD+ subgroup for JIA/ACR50, JIA/ACR70, and JIA/ACR‐CID. Efficacy outcomes with placebo tended to be more variable, depending on the use of concomitant MTX or prior bDMARDs. This is in line with a randomized trial of patients with pcJIA receiving subcutaneous golimumab (TNFi) treatment with background MTX, in which the primary outcome of JIA flare was not met, and background MTX therapy may have helped maintain disease control.^28^

In a previous trial of patients with pcJIA treated with the interleukin‐6 inhibitor, tocilizumab, for up to two years, numerically higher JIA/ACR70 and JIA/ACR90 response rates were reported by patients who were bDMARD‐naive compared with patients who were bDMARD‐experienced.^29^ Similarly, in the trial of the T cell costimulation modulator, abatacept, higher JIA/ACR70 and JIA/ACR90 response rates were reported in TNFi‐naive compared with TNFi‐experienced patients with pcJIA after four months of open‐label treatment (lead‐in phase).^11^ Together, these results are consistent with the results in this analysis of tofacitinib for JIA/ACR50, JIA/ACR70, and JIA/ACR‐CID rates. However, the differences in these outcomes between patients in the bDMARD+/− subgroups with tofacitinib treatment were not as pronounced as the differences between patients in the bDMARD subgroups with tocilizumab or abatacept treatments. In addition, with tocilizumab, numerically higher JIA/ACR70 and JIA/ACR90 responses were achieved in patients who received concomitant MTX treatment than in patients who did not receive concomitant MTX.^29^ Similar observations regarding concomitant MTX therapy were made in a Phase 3 withdrawal trial of patients with pcJIA receiving adalimumab (TNFi) with or without concomitant MTX. Compared with patients who did not receive concomitant MTX, numerically higher JIA/ACR50 and JIA/ACR70 responses were generally observed in patients who received concomitant MTX, although the trial design limited this interpretation.^30^ Head‐to‐head studies would be required to fully understand differences in efficacy between advanced treatments for JIA.^31^

In the analysis presented here, across outcomes, tofacitinib efficacy was generally similar between patients in the MTX+ and MTX− subgroups. However, because flare rates by Week 44 with placebo following tofacitinib withdrawal were greater for patients in the MTX− than in the MTX+ subgroup, these studies suggest that there may be a protective effect of MTX in preventing disease flare if advanced therapies are withdrawn, which should be considered when a patient is a candidate for drug discontinuation because of the achievement of inactive disease or clinical remission or if treatment modification is necessary for the patient due to AEs. Reduced efficacy of tofacitinib therapy in patients with prior bDMARD treatment has been reported in other disease indications such as rheumatoid arthritis and ankylosing spondylitis,^32^, ^33^ which, when taken together with observations with other advanced therapies, may imply that this population is more resistant to treatment.^10^, ^11^ It is important to note that patients in the bDMARD− subgroup had shorter disease duration at trial baseline compared with patients in the bDMARD+ subgroup, suggesting a window of opportunity earlier in the disease course wherein greater treatment responses with tofacitinib may be possible. It should also be noted that over 90% of patients in the trial had previously received MTX treatment for JIA; this was as expected because patients eligible for this trial were required to have an inadequate response to DMARD treatments (MTX or bDMARD).^14^ Further studies are warranted to improve our understanding of the underlying mechanisms of treatment responses to specific patient populations of JIA.

AEs and discontinuations because of AEs were more common in patients in the MTX− subgroup than the MTX+ subgroup and patients in the bDMARD+ subgroup than in the bDMARD− subgroup. This may in part be because of the higher rates of AEs associated with flares that were observed in patients without concomitant MTX use and/or with prior bDMARD exposure. Specifically, patients with prior bDMARD exposure may exhibit more refractory disease. Abnormalities in elevations of AST and ALT levels and low lymphocyte counts were more frequent in patients receiving tofacitinib with MTX compared with patients who did not receive MTX. These abnormalities in AST and ALT levels have previously been observed to be more frequent in patients with rheumatoid arthritis receiving tofacitinib with concomitant MTX use than tofacitinib monotherapy.^34^

Our study is not without potential limitations. This was a post hoc analysis and the number of patients in each of the subgroups was relatively low. Because data were as observed, the number of evaluable patients was reduced further for certain outcomes such as JADAS and CHAQ‐DI at Week 44. Data were not analyzed by oral glucocorticoid (prednisone) use. No formal statistical hypothesis testing was performed between the subgroups in each treatment arm. Therefore, differences that may be clinically important may provide opportunities for future hypothesis testing in a randomized controlled trial.

In patients with JIA, treatment with tofacitinib was efficacious irrespective of concomitant MTX treatment or prior bDMARD exposure. Following tofacitinib withdrawal, flare rates were lower, and there was a longer time to flare in patients treated with placebo with concomitant MTX treatment versus those without, suggesting MTX may have a protective effect in patients for whom it may be appropriate to withdraw tofacitinib. Safety was consistent with the known tofacitinib profile, and no new safety risks were identified with tofacitinib treatment in these subgroups.

## AUTHOR CONTRIBUTIONS

All authors contributed to at least one of the following manuscript preparation roles: conceptualization AND/OR methodology, software, investigation, formal analysis, data curation, visualization, and validation AND drafting or reviewing/editing the final draft. As corresponding author, Prof Ruperto confirms that all authors have provided the final approval of the version to be published and takes responsibility for the affirmations regarding article submission (eg, not under consideration by another journal), the integrity of the data presented, and the statements regarding compliance with institutional review board/Declaration of Helsinki requirements.

## ROLE OF THE STUDY SPONSOR

This study was sponsored by Pfizer. The study was designed jointly by Pfizer and the PRINTO/PRCSG officers (N Ruperto, DJ Lovell, HI Brunner, and A Martini). Pfizer was responsible for the overall management of the study and the data analysis. Publication of this article was not contingent upon approval by Pfizer.

## Supporting information


**Data S1:** CONSORT 2010 checklist of information to include when reporting a randomised trial

**Supplementary Figure 1:** JIA flare rate (SE) by Week 44 in patients receiving tofacitinib^a^ or placebo stratified by additional subgroups of concomitant MTX use or prior bDMARD exposure.
**Supplementary Figure 2:** Response rates (SE) over time in patients receiving tofacitinib^a^ or placebo stratified by A) concomitant MTX use and B) prior bDMARD exposure for JIA/ACR50 and JIA/ACR‐CID.
**Supplementary Figure 3:** Response rates (SE) over time in patients receiving tofacitinib^a^ or placebo stratified by additional subgroups of concomitant MTX use and prior bDMARD exposure for A) JIA/ACR50, B) JIA/ACR70 and C) JIA/ACR‐CID.
**Supplementary Figure 4:** Mean (SE) change from baseline^a^ in CHAQ‐DI at Week 44 in patients receiving tofacitinib^b^ or placebo stratified by additional subgroups of patients receiving concomitant MTX use and prior bDMARD exposure.
**Supplementary Table 1:** Safety events in the entire tofacitinib^a^ exposure period (Part 1 and Part 2) stratified by additional patient subgroups

**Disclosure Form**:

## APPENDIX A PRINTO AND PRCSG STUDY GROUP AUTHORS

Members of the PRINTO Study Group are as follows: N Ruperto, O Synoverska, C Abud‐Mendoza, A Spindler, Y Vyzhga, L Grebenkina, I Tirosh, B Sozeri, E Zholobova, and A Martini.

Members of the PRCSG Study Group are as follows: DJ Lovell, I Reyhan, L Imundo, P Chiraseveenuprapund, DJ Kingsbury, SS Vora, S Prahalad, and HI Brunner.
