# Supplementary material for: Impact of Concomitant Methotrexate Use and Prior Biologic Disease‐Modifying Antirheumatic Drug Exposure on Tofacitinib Efficacy and Safety in Patients with Polyarticular Course Juvenile Idiopathic Arthritis: Post Hoc Analysis of a Phase 3 Randomized Withdrawal Trial
Source: ACR Open Rheumatol. 2025 Oct 7;7(10):e70097. doi: 10.1002/acr2.70097 (PMC12504799; doi:10.1002/acr2.70097)
Supplement: Supplementary file 2 — Supplementary Figure 1: JIA flare rate (SE) by Week 44 in patients receiving tofacitiniba or placebo stratified by additional subgroups of concomitant MTX use or prior bDMARD exposure. Supplementary Figure 2: Response rates (SE) over time in patients receiving tofacitiniba or placebo stratified by A) concomitant MTX use and B) prior bDMARD exposure for JIA/ACR50 and JIA/ACR‐CID. Supplementary Figure 3: Response rates (SE) over time in patients receiving tofacitiniba or placebo stratified by additional subgroups of concomitant MTX use and prior bDMARD exposure for A) JIA/ACR50, B) JIA/ACR70 and C) JIA/ACR‐CID. Supplementary Figure 4: Mean (SE) change from baselinea in CHAQ‐DI at Week 44 in patients receiving tofacitinibb or placebo stratified by additional subgroups of patients receiving concomitant MTX use and prior bDMARD exposure. Supplementary Table 1: Safety events in the entire tofacitiniba exposure period (Part 1 and Part 2) stratified by additional patient subgroups [file ACR2-7-e70097-s001.docx]

# SUPPLEMENTARY MATERIALS

**Supplementary Figure 1.** JIA flare rate (SE) by Week 44 in patients receiving tofacitinib^a^ or placebo stratified by additional subgroups of concomitant MTX use or prior bDMARD exposure.

^
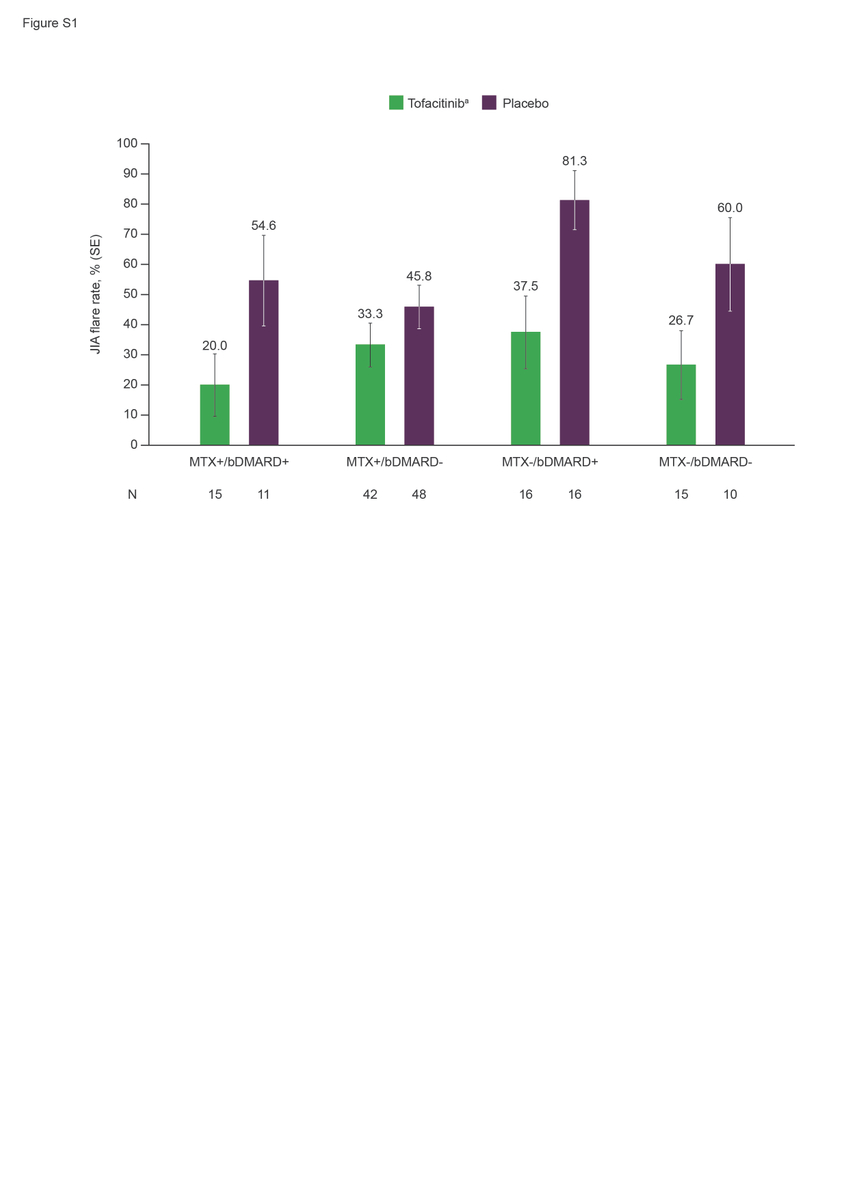
^
^a^Tofacitinib 5 mg BID or equivalent weight-based lower dose in patients weighing <40 kg.
bDMARD, biologic disease-modifying antirheumatic drug; BID, twice daily; JIA, juvenile idiopathic arthritis; MTX, methotrexate; N, number of evaluable patients; SE, standard error.

**Supplementary Figure 2.** Response rates (SE) over time in patients receiving tofacitinib^a^ or placebo stratified by A) concomitant MTX use and B) prior bDMARD exposure for JIA/ACR50 and JIA/ACR-CID.


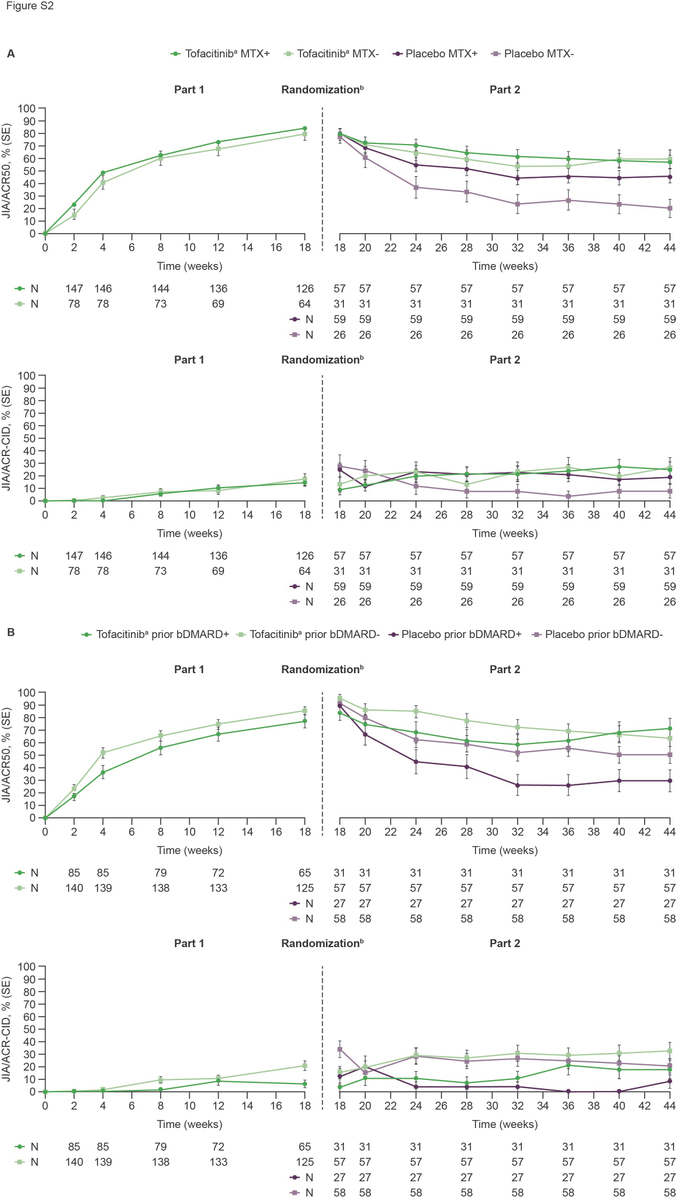


^a^Tofacitinib 5 mg BID or equivalent weight-based lower dose in patients weighing <40 kg. ^b^In Part 1
(Day 1–Week 18), all patients received open-label tofacitinib. Patients achieving ≥JIA/ACR30 response at Week 18 were blindly randomized 1:1 to receive tofacitinib or placebo in Part 2 (Weeks 18–44). Patients who discontinued during Part 2 due to flare or any other reason except for clinical remission (≥24 consecutive weeks of inactive disease), were considered to have active disease and classified as non-responders, as per the intention-to-treat principle^1-3^.
bDMARD, biologic disease-modifying antirheumatic drug; BID, twice daily; JIA/ACR50, juvenile idiopathic arthritis/American College of Rheumatology 50 response; JIA/ACR-CID, juvenile idiopathic arthritis/American College of Rheumatology clinical inactive disease, MTX, methotrexate; N, number of evaluable patients; SE, standard error.

**Supplementary Figure 3.** Response rates (SE) over time in patients receiving tofacitinib^a^ or placebo stratified by additional subgroups of concomitant MTX use and prior bDMARD exposure for A) JIA/ACR50, B) JIA/ACR70 and C) JIA/ACR-CID.


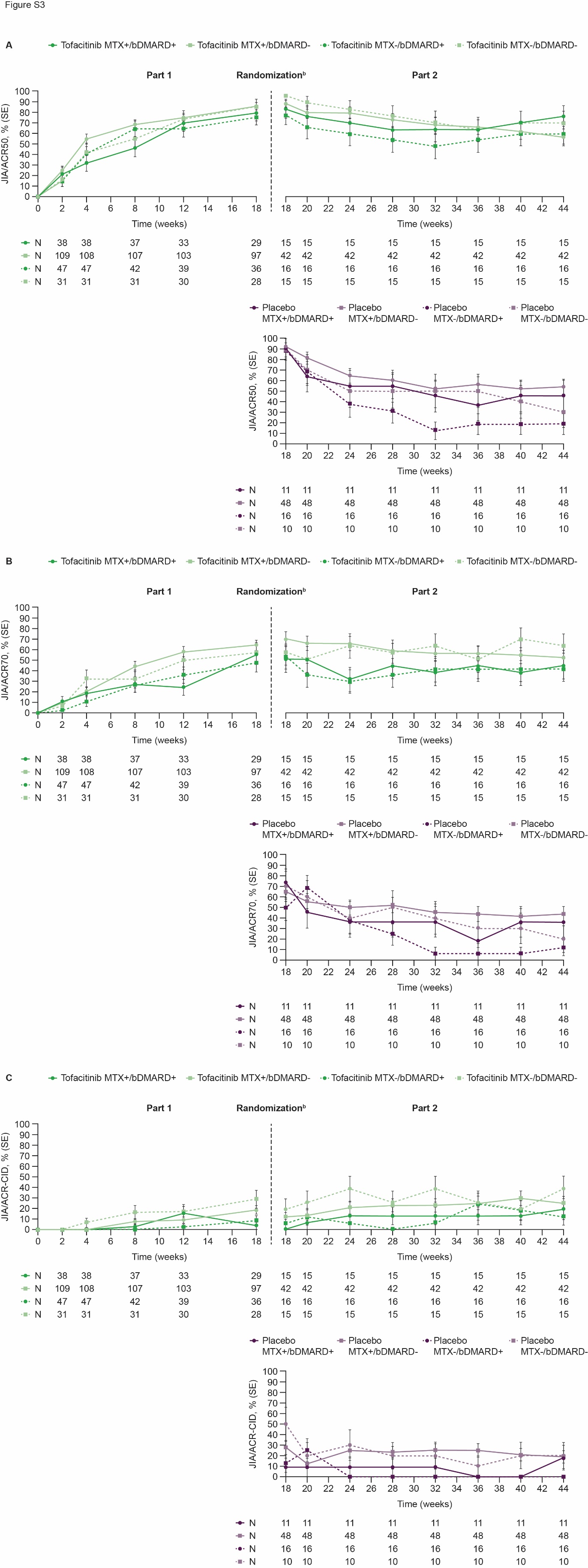


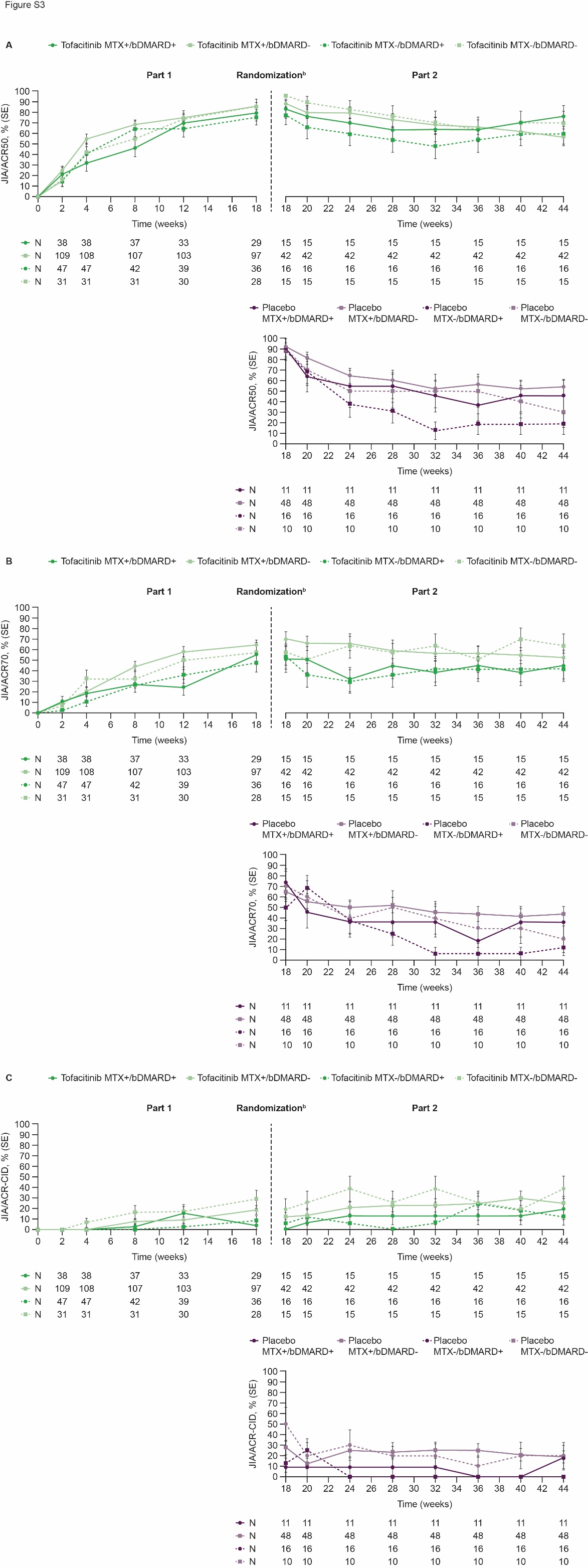


^a^Tofacitinib 5 mg BID or equivalent weight-based lower dose in patients weighing <40 kg. ^b^In Part 1
(Day 1–Week 18), all patients received open-label tofacitinib. Patients achieving ≥JIA/ACR30 response at Week 18 were blindly randomized 1:1 to receive tofacitinib or placebo in Part 2 (Weeks 18–44). Patients who discontinued during Part 2 due to flare or any other reason except for clinical remission (≥24 consecutive weeks of inactive disease), were considered to have active disease and classified as non-responders, as per the intention-to-treat principle^1-3^.
bDMARD, biologic disease-modifying antirheumatic drug; BID, twice daily; JIA/ACR50/70, juvenile idiopathic arthritis/American College of Rheumatology 50/70 response; JIA/ACR-CID, juvenile idiopathic arthritis/American College of Rheumatology clinical inactive disease; MTX, methotrexate; N, number of evaluable patients; SE, standard error.

**Supplementary Figure 4.** Mean (SE) change from baseline^a^ in CHAQ-DI at Week 44 in patients receiving tofacitinib^b^ or placebo stratified by additional subgroups of patients receiving concomitant MTX use and prior bDMARD exposure.


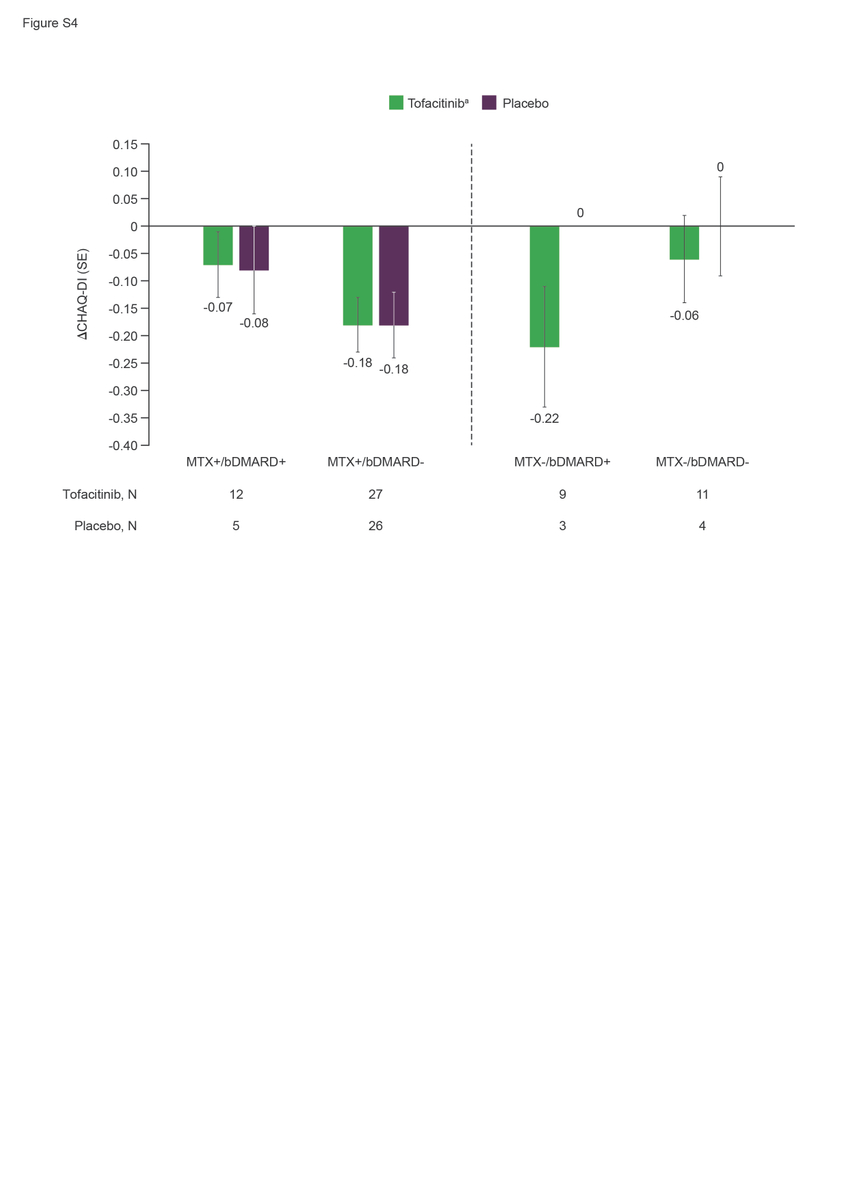


^a^Week 18 was used as baseline. ^b^Tofacitinib 5 mg BID or equivalent weight-based lower dose in patients weighing <40 kg.
ΔCHAQ-DI, change from baseline in Childhood Health Assessment Questionnaire – Disability Index; bDMARD, biological disease-modifying antirheumatic drug; BID, twice daily; MTX, methotrexate; N, number of evaluable patients; SE, standard error.

**Supplementary Table 1.** Safety events in the entire tofacitinib^a^ exposure period (Part 1 and Part 2) stratified by additional patient subgroups

| Patients with events, n (%) | MTX+/bDMARD+  (N = 38) | MTX+/bDMARD-  (N = 109) | MTX-/bDMARD+  (N = 47) | MTX-/bDMARD-  (N = 31) |
| --- | --- | --- | --- | --- |
| AEs | 29 (76.3) | 80 (73.4) | 40 (85.1) | 27 (87.1) |
| Serious AEs | 2 (5.3) | 3 (2.8) | 2 (4.3) | 1 (3.2) |
| Infections and infestations | 17 (44.7) | 48 (44.0) | 24 (51.1) | 18 (58.1) |
| Deaths | 0 | 0 | 0 | 0 |
| Permanent discontinuation due to AEs | 8 (21.1) | 15 (13.8) | 13 (27.7) | 6 (19.4) |
| Temporary dose reductions or temporary hold due to AEs | 4 (10.5) | 13 (11.9) | 4 (8.5) | 4 (12.9) |
| Most common AEs by preferred term (>5% of any subgroup) |  |  |  |  |
| Abdominal pain | 2 (5.3) | 2 (1.8) | 3 (6.4) | 1 (3.2) |
| Abdominal pain upper | 2 (5.3) | 1 (0.9) | 1 (2.1) | 1 (3.2) |
| Anemia | 0 | 3 (2.8) | 3 (6.4) | 0 |
| Arthralgia | 2 (5.3) | 2 (1.8) | 1 (2.1) | 2 (6.5) |
| Back pain | 1 (2.6) | 2 (1.8) | 2 (4.3) | 3 (9.7) |
| Condition aggravated | 0 | 1 (0.9) | 3 (6.4) | 1 (3.2) |
| Contusion | 2 (5.3) | 2 (1.8) | 0 | 0 |
| Cough | 2 (5.3) | 4 (3.7) | 2 (4.3) | 1 (3.2) |
| Decreased appetite | 0 | 1 (0.9) | 1 (2.1) | 4 (12.9) |
| Diarrhea | 2 (5.3) | 0 | 1 (2.1) | 3 (9.7) |
| Disease progression | 3 (7.9) | 4 (3.7) | 5 (10.6) | 1 (3.2) |
| Ear pain | 3 (7.9) | 0 | 0 | 0 |
| Epistaxis | 3 (7.9) | 0 | 2 (4.3) | 1 (3.2) |
| Gastroenteritis | 0 | 3 (2.8) | 1 (2.1) | 2 (6.5) |
| Hematuria | 2 (5.3) | 1 (0.9) | 0 | 0 |
| Headache | 1 (2.6) | 7 (6.4) | 5 (10.6) | 5 (16.1) |
| Increased ALT | 3 (7.9) | 5 (4.6) | 1 (2.1) | 0 |
| Increased AST | 3 (7.9) | 6 (5.5) | 1 (2.1) | 1 (3.2) |
| Increased blood creatine phosphokinase | 0 | 3 (2.8) | 1 (2.1) | 3 (9.7) |
| Influenza | 0 | 7 (6.4) | 3 (6.4) | 0 |
| JIA worsening | 2 (5.3) | 3 (2.8) | 2 (4.3) | 2 (6.5) |
| Nasopharyngitis | 3 (7.9) | 5 (4.6) | 4 (8.5) | 3 (9.7) |
| Nausea | 3 (7.9) | 10 (9.2) | 0 | 1 (3.2) |
| Oropharyngeal pain | 1 (2.6) | 1 (0.9) | 3 (6.4) | 1 (3.2) |
| Pyrexia | 1 (2.6) | 7 (6.4) | 3 (6.4) | 3 (9.7) |
| Pharyngitis | 4 (10.5) | 1 (0.9) | 0 | 2 (6.5) |
| Respiratory tract infection | 0 | 5 (4.6) | 0 | 1 (3.2) |
| Sinusitis | 0 | 4 (3.7) | 2 (4.3) | 2 (6.5) |
| Vomiting | 2 (5.3) | 5 (4.6) | 3 (6.4) | 3 (9.7) |
| Upper respiratory tract infection | 1 (2.6) | 17 (15.6) | 9 (19.1) | 7 (22.6) |
| Urinary tract infection | 0 | 3 (2.8) | 0 | 2 (6.5) |
| Laboratory abnormalities |  |  |  |  |
| AST >3.0 × ULN | 1 (2.6) | 3 (2.8) | 0 | 0 |
| ALT >3.0 × ULN | 1 (2.6) | 5 (4.6) | 0 | 0 |
| Haemoglobin <0.8 × LLN | 1 (2.7)^b^ | 1 (0.9) | 0 | 0 |
| Lymphocytes |  |  |  |  |
| <0.8 × LLN | 1 (2.7)^b^ | 10 (9.2) | 0 | 0 |
| >1.2 × ULN | 2 (5.4)^b^ | 1 (0.9) | 0 | 0 |

^a^Tofacitinib 5 mg BID or equivalent weight-based lower dose in patients weighing <40 kg. ^b^N = 37.
AE, adverse event; AESI, adverse events of special interest; ALT, alanine aminotransferase; AST, aspartate aminotransferase; bDMARD, biologic disease-modifying antirheumatic drug; BID, twice daily; JIA, juvenile idiopathic arthritis; LLN, lower limit of normal; MTX, methotrexate; N, number of evaluable patients; n, number of patients with events; ULN, upper limit of normal.

# REFERENCES

1. Schulz KF, Altman DG, Moher D, et al. CONSORT 2010 Statement: updated guidelines for reporting parallel group randomised trials. BMC Med 2010;8:18.

2. Moher D, Schulz KF, Altman DG. The CONSORT statement: revised recommendations for improving the quality of reports of parallel-group randomised trials. Lancet 2001;357:1191-1194.

3. Hopewell S, Chan A-W, Collins GS, et al. CONSORT 2025 statement: updated guideline for reporting randomised trials. Lancet 2025; 405:1633–1640.
